# A large ectopic hepatocellular carcinoma with adrenal infiltration: a rare case report

**DOI:** 10.3389/fonc.2023.1116684

**Published:** 2023-04-24

**Authors:** Yongjun Yang, Qiang Lu, Zonglin Li, Chen Wang, Yuanwei Li

**Affiliations:** Department of Urology, Hunan Provincial People’s Hospital, The First Affiliated Hospital of Hunan Normal University, Changsha, Hunan, China

**Keywords:** ectopic hepatocellular carcinoma, retroperitoneal tumor, adrenal infiltration, endocrine function, case report

## Abstract

Ectopic hepatocellular carcinoma (EHCC) originates from the ectopic liver, which refers to a liver organ or tissue unrelated to surrounding tissues. EHCC is a rare disease that lacks specific clinical signs, and preoperative diagnosis is often difficult. In a 61-year-old male patient with positive hepatitis B virus antibody, abdominal contrast-enhanced computed tomography scan showed a large heterogenously enhancing mass both on arterial and portal venous phase imaging arising from the right adrenal gland. Similar enhancement features were seen on magnetic resonance imaging. Serum potassium, aldosterone, cortisol, and plasma metanephrines were normal. The tumor markers of serum alpha-fetoprotein and alpha-fetoprotein-L3% were increased to 23.69 ng/mL and 82.1%, respectively. Exploratory laparotomy was performed and operative findings showed that the retroperitoneal tumor was disconnected from the right kidney and the liver, but invaded the right adrenal gland. Immunohistochemical examination showed that Arginase-1 was positive expression, and the retroperitoneal tumor was finally diagnosed as EHCC. We report a rare EHCC with adrenal infiltration that is difficult to diagnose preoperatively and mimics a retroperitoneal tumor or adrenal tumor, and we present a review of the literature on EHCC case reports.

## Introduction

Ectopic hepatocellular carcinoma (EHCC) is defined as hepatocellular carcinoma arising from the hepatic parenchyma located in an extrahepatic organ or tissue (1). It is reported that the incidence of ectopic liver during laparoscopy or autopsy ranges from 0.24% to 0.47% (2). Ectopic liver can be located in various organs or tissues near the liver, such as gallbladder, adrenal gland, pancreas, peritoneum and thorax (3–6). Due to the variable location, the clinical signs of EHCC are still not fully elucidated. Thus, EHCC is a very rare tumor disease with variable location and lack of specific clinical signs, which increases the difficulty of accurate preoperative diagnosis. Here, we report a case of EHCC with adrenal infiltration mimicking a retroperitoneal tumor or adrenal tumor and review the literature concerning EHCC.

## Case presentation

The case was a 61-year-old male patient with positive hepatitis B virus antibody (HBV-DNA was 2.174 × 10^2^), and he presented with abdominal distention and hyporexia for one week. Due to abdominal distension and hyporexia, the patient went to a nearby outpatient clinic, and abdominal ultrasound examination showed a large solid mass with mixed echogenicity. The patient had no history of hypertension, diabetes, cardiovascular and cerebrovascular diseases, and no history of surgery. His abdomen was soft and flat. There was mild tenderness in the right upper quadrant, but Murphy’s sign was negative. The liver was palpable 2 cm below the costal margin due to the push of the mass. The patient lost 5 kg in weight last month. (Eastern Cooperative Oncology Group: 0).

**Figure 1:**
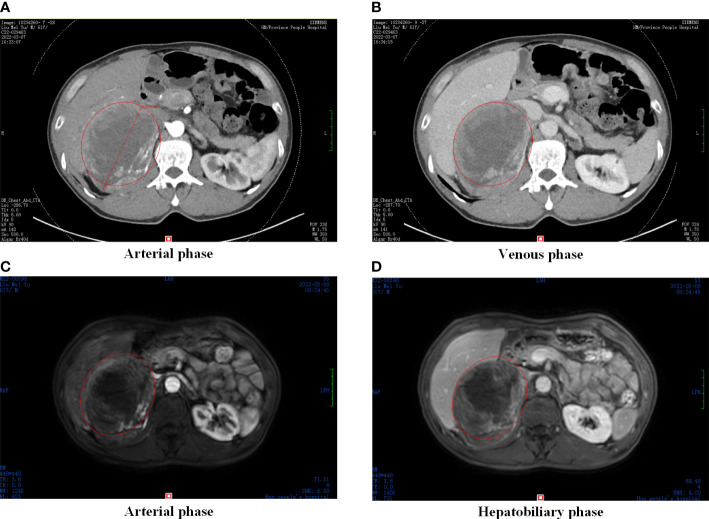
Preoperative abdominal CT and MRI. **(A)** CT scan in arterial phase, **(B)** CT scan in venous phase; **(C)** MRI scan in arterial phase, **(D)** MRI scan in hepatobiliary phase. Abdominal CT and MRI images showed an oval and smoothly mass located in the liver-kidney space (red circle).

Abdominal contrast-enhanced computed tomography (CT) scan showed that the retroperitoneal tumor (11.8 × 11.0 × 8.3 cm) located in the right adrenal region, with obvious enhancement in arterial phase and continuous enhancement in venous phase (**Figures 1A, B**). Magnetic resonance imaging (MRI) displayed uneven enhancement in arterial phase and no abnormal signal focus in hepatobiliary phase (**Figures 1C, D**). On the laboratory tests, because the tumor was located in the adrenal region, serum potassium, aldosterone, cortisol, and plasma metanephrines were tested, and the results were normal. Serum alpha-fetoprotein (AFP) and AFP-L3% were 23.69 ng/mL and 82.1%, respectively.

**Figure 2:**
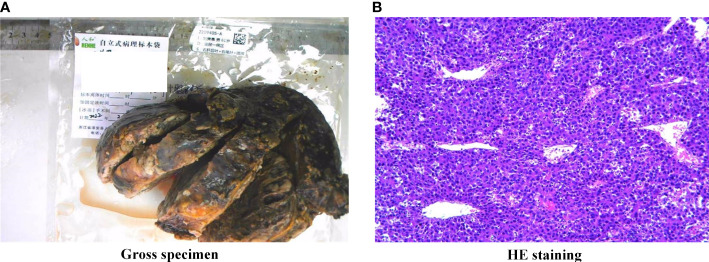
Gross specimen and HE staining. **(A)** Macroscopic features of the excised hepatocellular carcinoma demonstrating a solid tumor with a fibrous capsule and intratumoral hemorrhage, **(B)** HE staining of the tumor confirmed poorly differentiated carcinoma morphologically, magnification × 100. The hepatoma cells were large, polygonal, rich in cytoplasm and red staining, and were diagnosed as massive beam and cord liver cancer.

**Figure 3:**
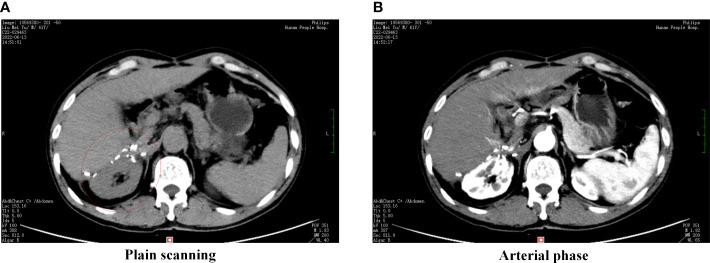
Postoperative abdominal CT. **(A)** CT scan in plain scanning, **(B)** CT scan in arterial phase. Postoperative abdominal CT images showed no new mass in the liver-kidney space (red circle).

The patient underwent an exploratory laparotomy to remove the tumor from an unknown primary organ in the adrenal region. On exploration, there was a large encapsulated oval solid tumor occupying the right retroperitoneal cavity, adjacent to the liver and right kidney, and infiltrating the adrenal tissue. The blood supply of the tumor is supplied by small branches of the right renal artery and abdominal aorta, and the adjacent liver, right kidney, and inferior vena cava are compressed and displaced by the tumor. The gross size of the resected specimen was about 16.0 × 14.0 × 10.0 cm and encapsulated in membrane, and the cut surface was reddish-yellow with intratumoral hemorrhage. Histopathological examination of tumor specimen showed that there were a large number of hepatoma cells arranged in beam, cord and nest shapes (**Figure 2**). Golden yellow adrenal tissue can be seen on the surface of the mass, and the pathological results suggest that cancer cells invade the adrenal gland. Pathological examination of the tumor specimen did not reveal any significant histological structure of the biliary tract. Therefore, it is speculated that ectopic hepatocytes may not have normal bile secretion functions, and this variation contributes to the degeneration or even canceration of hepatocytes. On immunohistochemical staining of the tumor specimen, Hepatocyte and Glypican-3 were negative and focal positive, respectively, but Arginase-1 (Arg-1) was positive. Moreover, GS-6 was positive, Ki67 was partly positive, Inhibin-a was negative, S-100 was negative, p53 was negative, and Melan-A was negative (**Table 1**). On postoperative laboratory tests, the levels of AFP and AFP-L3% had decreased to 5.98 ng/mL and 35.4%, respectively. Finally, we diagnosed the retroperitoneal tumor as EHCC. After the diagnosis of EHCC by histopathological examination, the patient received adjuvant treatment with the PD-1 blocker sintilimab and lenvatinib. Twelve months after operation, abdominal contrast-enhanced CT scan showed postoperative changes, and no tumor recurrence was found (**Figure 3**). Currently, twelve months have passed, the patient is still alive without recurrence.

**Table 1:** Details of immunohistochemical staining of the resected tumor specimen.

Variables	Results
CK7	Positive
CK19	Positive
CD10	Positive
CD34	Positive
Glypican-3	Focal positive
GS-6	Positive
Hepatocyte	Negative
Arg-1	Positive
Syn	Negative
CgA	Negative
p53	Negative
Ki67	Positive (80%)
S-100	Negative
Melan-A	Negative
Inhibin-a	Negative
SF-1	Negative

## Discussion

EHCC is a rare carcinomas defined as an HCC arising from ectopic liver organ or tissue. The incidence rate of ectopic liver is about 0.24% to 0.47%. When we searched for “ectopic hepatocellular carcinoma” as a “Text Word” in PubMed, only 28 full-text case reports written in English were obtained (**Table 2**) (1, 3–29). Ectopic liver tissue can occur in the adrenal gland, pancreas, peritoneum, gallbladder, bile duct, thoracic cavity, abdominal cavity, diaphragm, spleen, and chest wall, of which the diaphragm is the most common site (12, 13, 16, 20, 24, 27, 29).

**Table 2:** Literature review of ectopic hepatocellular carcinoma (EHCC).

Case	Study	Age (year)	Sex	Size (cm)	Number	Location	Virus infection	AFP (ng/mL)	Metastasis	Treatment	Follow-up (months)	Outcome
1	Our case/2022	61	M	11.8×11.0 ×8.3	Single	Right adrenal	HBV	23.69	No	Laparotomy + Lenvatinib	6	No Recurrence
2	Liu et al. (1)	59	M	1.8×1.4	Single	Tail of pancreas	Negative	3.1	No	Laparotomy	84	No Recurrence
3	Wei et al. (3)	71	M	9.1×8.2×8.6	Single	Right adrenal	Negative	Normal	No	Laparotomy	10	Recurrence
4	Adachi et al. (4)	81	F	6.0	Single	Head of pancreas	HCV	30.1	No	Laparotomy	8	No Recurrence
5	Ko et al. (5)	73	M	5.5	Multiple	Peritoneum	Negative	1.164	Yes	Laparoscopy + Sorafenib	12	Recurrence and died
6	Rorris et al. (6)	53	M	3.2	Single	Right adrenal	HCV	Normal	Yes	Laparotomy	N/A	No Recurrence
7	Li et al. (7)	44	F	5.0 × 4.0	Multiple	Near the pancreas	Negative	553.90	No	Laparotomy	17	No Recurrence
8	Jin et al. (8)	56	M	30.0 × 20.0 × 15.0	Multiple	Abdominal	Negative	8.03	Yes	Laparotomy	22	Recurrence and died
9	George et al. (9)	69	M	N/A	Single	Choledochal cyst	Negative	2.90	No	Refuse resection	N/A	N/A
10	Cheng et al. (10)	54	M	N/A	Single	Bile duct and gallbladder	HBV	3724.75	No	Laparotomy	3	No Recurrence
11	Cui et al. (11)	63	M	4.6 × 2.2	Multiple	Thoracic and abdominal cavities	HBV	24,793	Yes	Palliative surgery + Sorafenib	13	Progression free survival
12	Lee et al. (12)	65	M	3.8×3.2×1.2	Single	Left subphrenic region	Negative	Normal	No	Surgical resection	17	No Recurrence
13	Aarås et al. (13)	64	F	3.5×2.5×1.0	Single	Diaphragm	Negative	200	No	Laparoscopy	48	Recurrence
14	Segura-Sánchez et al. (14)	49	F	12.0	Single	Gallbladder	Negative	13,785	No	Laparotomy	36	No Recurrence
15	Miyake et al. (15)	42	M	1.0	Multiple	Abdominal cavity	Negative	241	Yes	Laparoscopy + chemotherapy	N/A	N/A
16	Nishikawa et al. (16)	64	M	N/A	Multiple	Left diaphragm	Negative	84,865	Yes	N/A	N/A	N/A
17	Nenekidis et al. (17)	68	M	7.0	Multiple	chest wall and skull	Negative	Elevated	Yes	Surgical resection	24	No Recurrence
18	Singh et al. (18)	60	M	8.0×8.0×8.0	Single	Left suprarenal region	HBV	35	No	Laparotomy	6	Recurrence and died
19	Matsuyama et al. (19)	69	M	6.0×4.0	Multiple	Spleen	N/A	N/A	No	Chemotherapy	25	Recurrence and died
20	Kanzaki et al. (20)	59	F	2.0	Single	Left diaphragm	Negative	2,508	No	Laparoscopy	18	No Recurrence
21	Schmelzle et al. (21)	75	M	N/A	Single	Extrahepatic bile duct	N/A	7.8	No	Laparotomy	N/A	N/A
22	Seo et al. (22)	59	M	4.5	Single	Left subphrenic space	Negative	Normal	No	Laparoscopy	N/A	N/A
23	Kubota et al. (23)	56	M	6.3×6.2	Single	Tail of pancreas	Negative	N/A	No	Laparotomy	36	No Recurrence
24	Huang et al. (24)	62	F	16.0×14.0×5.0	Single	Diaphragm	Negative	45,000	No	Laparotomy + chemotherapy	8	No Recurrence
25	Shigemori et al. (25)	72	M	14.0×10.0	Single	Lower abdomen	Negative	99,100	No	Laparotomy + TACE	12	No Recurrence
26	Tsushimi et al. (26)	72	F	2.7×2.1	Single	Bile duct	Negative	N/A	No	Laparotomy	12	No Recurrence
27	Kim et al. (27)	43	F	10.0×7.0	Single	Between spleen and diaphragm	HBV	N/A	No	Laparotomy + TACE	23	Recurrence
28	Asselah et al. (28)	66	M	17.0×10.0×8.0	Single	Left chest wall	HCV	Normal	No	Surgical resection	36	No Recurrence
29	Takayasu et al. (29)	57	M	5.0×4.0	Single	Left diaphragm	Negative	2,207	No	Laparotomy	96	No Recurrence

HBV, hepatitis B virus; HCV, hepatitis C virus; N/A, none of available; TACE, transcatheter arterial chemoembolization.

Of the 29 patients including this case, 26 patients underwent surgery treatment (1, 3–8, 10–15, 17, 18, 20–29) and two patients did not undergo surgery due to serious complications (9, 19). Traditional laparotomy and laparoscopy can be performed as the surgical methods, and seven patients received adjuvant therapy after surgery (5, 11, 15, 24, 25, 27). For unresectable hepatocellular carcinoma, lenvatinib or sorafenib can be used as targeted drugs for adjuvant therapy in EHCC (30). After surgical resection alone or combined with adjuvant therapy, EHCC patients have a relatively good oncological prognosis, with 7/23 (30.4%) patients experiencing tumor recurrence. Therefore, if the patient does not have surgical contraindications, surgery should be recommended first. During follow-up, the clinical features of the tumors in the dead patients were multiple or relatively large in size. Thus, for patients with multiple or large tumors, a more rigorous follow-up plan should be developed to detect tumor recurrence early.

In previous case reports, three out of 28 tumors were located in the adrenal gland, but only one of them was located in the left adrenal gland region, while this case was located in the right adrenal gland region (3, 6, 18). Because the tumor is located in the adrenal region, it is difficult to diagnose EHCC before surgery, and it is easy to be misdiagnosed as an adrenal tumor. The tumor in this case invaded the adrenal tissue, which increased the difficulty of preoperative diagnosis. For EHCC located in the adrenal region, preoperative adrenal-related hormone testing is needed to determine whether the tumor has endocrine function. Except for one patient who did not undergo endocrine evaluation due to hemodynamic compromise and type I respiratory failure requiring emergency surgery, the other three patients underwent adrenal endocrine function evaluation. According to the test results, serum potassium, aldosterone, cortisol, and plasma metanephrines were normal, and the adrenal gland had no abnormal endocrine function (3, 6, 18).

## Conclusion

Preoperative diagnosis of EHCC is usually very difficult. The tumor located in the adrenal region further increase the difficulty of diagnosis, and it is easy to be misdiagnosed as an adrenal tumor. Adrenal-related hormone testing can be performed to evaluate tumor endocrine function if radiographic studies reveal a tumor in the adrenal region. Early surgical treatment of EHCC will provide good long-term outcomes.

## Patient perspective

His family is pleased that the patient is alive, and that the patient’s liver and kidney are protected during the operation. Both the patient and his families feel that if this exploratory laparotomy could ensure complete tumor resection and minimize damage to surrounding organs, they would like to choose such a surgical treatment strategy.

## Data availability statement

The original contributions presented in the study are included in the article/supplementary material. Further inquiries can be directed to the corresponding author.

## Ethics statement

The studies involving human participants were reviewed and approved by the Ethics Committee of Hunan Provincial People’s Hospital. The patients/participants provided their written informed consent to participate in this study. Written informed consent was obtained from the participant/patient(s) for the publication of this case report.

## Author contributions

Manuscript writing: YY and YL; Clinical case diagnosis and treatment: YY, QL, and YL; Data collection and literature resarch: YY, ZL, and CW; Manuscript review and revision: YY and QL. All authors contributed to the article and approved the submitted version.
